# Murine gammaherpesvirus-68 glycoprotein B presents a difficult neutralization target to monoclonal antibodies derived from infected mice

**DOI:** 10.1099/vir.0.82313-0

**Published:** 2006-12

**Authors:** Laurent Gillet, Michael B. Gill, Susanna Colaco, Christopher M. Smith, Philip G. Stevenson

**Affiliations:** Division of Virology, Department of Pathology, University of Cambridge, Tennis Court Road, Cambridge CB2 1QP, UK

## Abstract

Persistent viruses disseminate from immune hosts. They must therefore resist neutralization by antibody. Murine gammaherpesvirus-68 (MHV-68) represents an accessible model with which to address how resistance to neutralization is achieved and how overcoming it might improve infection control. The MHV-68 glycoprotein B (gB), like that of other herpesviruses, is a virion protein that is essential for infectivity. As such, it presents a potential neutralization target. In order to test whether virus-induced antibodies reduce virion infectivity by binding to gB, monoclonal antibodies (mAbs) were derived from MHV-68-infected mice. gB-specific mAbs were common, but only an IgM specific for the gB N terminus reduced virion infectivity significantly. It inhibited MHV-68 entry into BHK-21 cells at a post-binding step that was linked closely to membrane fusion. Reducing the mAb to IgM monomers compromised neutralization severely, suggesting that a pentameric structure was crucial to its function. Antibody treatment never blocked BHK-21 cell infection completely and blocked the infection of NMuMG epithelial cells hardly at all. Virions saturated with antibody also remained infectious to mice. Thus, the MHV-68 gB presents at best a very difficult target for antibody-mediated neutralization.

## INTRODUCTION

Herpesviruses are among the most successful of all vertebrate parasites. They robustly establish persistent, productive infections of immunocompetent hosts. This is achieved without generalized immunosuppression, tolerance of viral gene products or significant viral antigenic variation. For example, the murine cytomegalovirus glycoprotein B (gB) is immunogenic, yet the region associated with neutralization is antigenically conserved in field isolates (Xu *et al.*, 1996). Thus, infected individuals simultaneously produce virus-specific antibodies and secrete infectious virions. They also remain susceptible to reinfection (Klein, 1989; Boppana *et al.*, 2001; Sitki-Green *et al.*, 2003; Gorman *et al.*, 2006). A major infection-control challenge is to work out why established antibody responses fail to stop herpesviruses trafficking in or out of their hosts.

Many persistent viruses resist neutralization by antibody. Human immunodeficiency virus (HIV) (Pantophlet & Burton, 2006) demonstrates the difficulty of neutralizing even virions with just one glycoprotein species. Herpesviruses – which generally express at least 10 different virion glycoproteins – are more transmissible than HIV and must therefore resist neutralization at least as well. However, immune sera appear to neutralize herpesviruses *in vitro* (Fenner *et al.*, 1974). Some antibodies may therefore be capable, if present in sufficient amounts, of neutralizing herpesviruses *in vivo*. Immune sera are notoriously complex. Monoclonal antibodies (mAbs) provide a simpler way to identify key neutralization targets, and neutralizing mAbs derived from virus carriers are of particular interest. They represent responses that could potentially be boosted by vaccination to reduce long-term shedding of infectious virions.

Our focus is on the gammaherpesviruses. The major neutralization target defined for the archetypal gammaherpesvirus, Epstein–Barr virus (EBV), is gp350 (Thorley-Lawson & Poodry, 1982). However, gp350 is redundant for epithelial-cell infection (Janz *et al.*, 2000), raising questions as to the *in vivo* significance of this neutralization. Partial neutralization of Kaposi's sarcoma-associated herpesvirus (KSHV) has been reported for rabbit sera raised against recombinant gH, gL (Naranatt *et al.*, 2002) or gB (Akula *et al.*, 2002). Whether these specificities account for the *in vitro* neutralizing activity of human immune sera (Dialyna *et al.*, 2004) is unknown. Analyses of EBV and KSHV neutralization are hindered by the difficulty of growing these viruses *in vitro* and by the limited opportunities for manipulation *in vivo*. Murine gammaherpesvirus-68 (MHV-68) is a natural parasite of yellow-necked mice (Kozuch *et al.*, 1993) that addresses some of these problems (Stevenson & Efstathiou, 2005). Conventional mice also appear to behave as natural MHV-68 hosts, in that infection persists without causing disease, unless there is immune suppression. After intranasal infection, MHV-68 replicates lytically in respiratory epithelial cells and establishes latency in memory B cells (Sunil-Chandra *et al.*, 1992a; Flaño *et al.*, 2002). Immune sera from infected mice (Stevenson & Doherty, 1998, 1999) or from rabbits immunized with whole virus (Sunil-Chandra *et al.*, 1992b), gp150 (Stewart *et al.*, 1999) or ORF4 (Gangappa *et al.*, 2002) have all been reported to neutralize MHV-68 for fibroblast infection. However, neither ORF4 (Adler *et al.*, 2000) nor gp150 (de Lima *et al.*, 2004) is essential for infectivity. Comparison with other herpesviruses, as well as the general principle that robust neutralization should inactivate essential proteins, would suggest gH/gL and gB as more likely targets (Moorman *et al.*, 2004; Song *et al.*, 2005).

We recently derived several gH/gL-specific neutralizing mAbs from MHV-68-infected mice (Gill *et al.*, 2006). We did not find neutralizing mAbs specific for ORF4 or gp150. Surprisingly, we also failed to identify neutralizing mAbs specific for gB, even though gB is often reported as a herpesvirus neutralization target (Cranage *et al.*, 1986; Pereira *et al.*, 1989; Takeda *et al.*, 1996; Akula *et al.*, 2002; Wang *et al.*, 2003b). gB is generally essential for membrane fusion (Turner *et al.*, 1998; Pertel, 2002; Lopper & Compton, 2004). It often also has a receptor-binding/signalling function. For example, the human cytomegalovirus gB binds to the epidermal growth factor (EGF) receptor (Wang *et al.*, 2003b) and the KSHV gB binds to integrins (Akula *et al.*, 2002). Blocking either interaction inhibits infection at a post-binding step. The MHV-68 gB has a consensus heparin-binding sequence equivalent to that of the KSHV gB (Akula *et al.*, 2001), but lacks the RGD motif essential for KSHV integrin binding (Wang *et al.*, 2003a). Whether gB makes a significant contribution to MHV-68 cell binding is not known.

A possible reason for our previous failure to identify gB-specific, MHV-68-neutralizing mAbs is that we focused on only the most effective mAbs, regardless of their specificity. We have now looked more explicitly for gB-directed neutralization. This search led to the isolation of a gB-specific mAb capable of blocking infection at a post-binding step. However, gB-directed neutralization was far from universally effective. We present here an analysis of how a gB-specific neutralizing antibody interacts with MHV-68 virions.

## METHODS

### Mice.

Female BALB/c mice were purchased from Harlan UK Ltd, housed in the Cambridge University Department of Pathology and infected intranasally with MHV-68 when 6–12 weeks old. For mAb production, three mice were given an intraperitoneal virus boost at 3–6 months post-infection. Spleens were harvested and pooled 3 days later. To recover infectious virus, lungs were removed at 7 days post-infection, freeze–thawed and broken up by Dounce homogenization. Large debris was removed by centrifugation (200 ***g***, 1 min) and homogenates were stored at −70 °C.

### Viruses.

Fluorescent-tagged MHV-68 was made by attaching enhanced green fluorescent protein (EGFP) to the C terminus of gM (May *et al.*, 2005a). The gM coding sequence (genomic co-ordinates 56950–55799) was amplified (Hi-Fidelity PCR kit; Roche Diagnostics) by using an *Xho*I-restricted 5′ primer (5′-TATCTCGAGATGCCTGCCCTTAAAGTGCCA-3′) and a *Bam*HI-restricted 3′ primer (5′-TTTGGATCCTAGTTCATCTTCTGATTCTGTCTC-3′) and cloned into the *Bam*HI and *Xho*I sites of pEGFP-N3 (Clontech). This placed the EGFP coding sequence downstream of and in frame with that of gM. Genomic co-ordinates 55800–54649 of MHV-68 were then amplified with an *Afl*II-restricted 5′ primer (5′-AAACTTAAGAGCTCTAGCCTTTGGATAAGATGTGAA-3′) and a *Not*I-restricted 3′ primer (5′-AAAGCGGCCGCAAACATTTTATTAAAGTAATTAAAGCAAAT-3′) and cloned into the *Afl*II and *Not*I sites of pEGFP-N3-gM. The EGFP coding sequence with its 1150 bp MHV-68 genomic flanks was excised by using a *Bgl*II site in the pEGFP-N3 polylinker and the *Sac*I site in the *Afl*II-restricted PCR primer, ligated into the *Bam*HI and *Sac*I sites of pST76K-SR and recombined into an MHV-68 genomic bacterial artificial chromosome (BAC) by standard protocols (Adler *et al.*, 2000). Infectious virus was reconstituted by transfecting BAC DNA into BHK-21 cells with Fugene 6 (Roche Diagnostics). The *loxP*-flanked BAC cassette was removed by passaging virus through NIH-3T3-CRE cells (Stevenson *et al.*, 2002). Virus stocks were grown in BHK-21 cells (Coleman *et al.*, 2003).

### Plasmids.

The coding sequences for aa 25–423, 25–65 or 428–698 of the mature gB extracellular domain were cloned as *Eco*RI/*Xho*I- or *Eco*RI/*Sal*I-restricted PCR products (Phusion DNA polymerase; New England Biolabs) into the *Eco*RI and *Xho*I sites of pGEX-4T-1 (Amersham Biosciences). They were then expressed as fusions with N-terminal glutathione *S*-transferase (GST) by transformation into *Escherichia coli* BL21 and induction with IPTG (Boname *et al.*, 2005). The bacteria were lysed in 150 mM NaCl, 50 mM Tris (pH 7.4), 5 mM EDTA, 1 % Triton X-100 with Complete Protease inhibitors (Roche Diagnostics), 1 mM PMSF and 1 mM *N*-ethylmaleimide. GST fusion proteins were recovered by adsorption to glutathione–Sepharose beads (Amersham Biosciences), washed three times in lysis buffer and eluted with an excess of free glutathione. The MHV-68 ORF65 coding sequence was expressed as a GST fusion protein in a similar way. We have previously expressed the gB extracellular domain (genomic co-ordinates 16526–18617) as a glycosylphosphatidylinositol (GPI)-linked membrane protein (Lopes *et al.*, 2004). Amino acid residues 1–423 of gB were expressed similarly by amplifying the relevant region of the MHV-68 genome with 5′ and 3′ primers containing *Avr*II and *Not*I restriction sites, respectively, and cloning the PCR product into the *Xba*I/*Not*I sites of pBRAD. The coding sequence for aa 428–698 of gB was amplified by using a blunt-ended 5′ primer and a *Not*I-restricted 3′ primer. This PCR product was ligated into pBRAD-gB cut with *Not*I and *Sna*BI. This left in place the gB coding sequence up to aa 22, and the 5′ primer included the coding sequence for aa 23–25. Thus, aa 428–698 were linked to the normal gB signal sequence (residues 1–24) plus residue 25.

### Cells and antibodies.

BHK-21 cells, NIH-3T3 cells, NIH-3T3-CRE cells (Stevenson *et al.*, 2002), NS0 cells, MRC-5 cells, 293T cells, NMuMG cells and murine embryonic fibroblasts (MEFs) were propagated in Dulbecco's modified Eagle's medium supplemented with 2 mM glutamine, 100 U penicillin ml^−1^, 100 μg streptomycin ml^−1^, 50 μM 2-mercaptoethanol and 10 % fetal calf serum (May *et al.*, 2005b). B-cell hybridomas were generated by fusing splenocytes with NS0 cells using PEG 1500 (Roche Diagnostics). Hybrids were cultured on irradiated MRC-5 feeder cells and selected with azaserine (1 μg ml^−1^)/hypoxanthine (100 μM) (Gill *et al.*, 2006). mAbs were concentrated from hybridoma supernatants by ammonium sulfate precipitation, dialysed against PBS and quantified by ELISA against mouse immunoglobulin standards. Isotyping was by ELISA using isotype-specific capture and/or detection antibodies (Sigma). Where indicated, ammonium sulfate-precipitated mAbs were biotinylated with *N*-hydroxybiotin–succinamide ester (Pierce) and then dialysed against PBS to remove free biotin. All of the mAbs used are listed in Table 1. A biotinylated goat anti-GST polyclonal antibody (pAb) was purchased from Santa Cruz Biotechnology.

### Virus assays.

Infectious virus was titrated by plaque assay (Coleman *et al.*, 2003). Virus preparations were centrifuged (200 ***g***, 1 min) prior to neutralization assays to remove any large debris. Neutralization was tested by incubating virus with antibody for 1 h at 37 °C before adding the virus/antibody mixtures to cells for a further 2 h. The cells were then overlaid with 0.3 % carboxymethylcellulose and the cell monolayers were fixed and stained for plaque counting after a further 4–5 days. For binding/entry assays, virus was incubated with cells at 4 or 37 °C, then washed in PBS (pH 7.4) to remove unbound virus or in isotonic citrate buffer (pH 3.0) to remove any virus that had not penetrated the plasma membrane or been endocytosed. Infection was quantified by plaque assay, and virion uptake by immunofluorescence or flow cytometry.

### Immunoblotting.

Virions were recovered from infected-cell supernatants by ultracentrifugation (35 000 ***g***, 90 min) and denatured by heating in 0.1 % SDS/1 % 2-mercaptoethanol (95 °C, 5min). Where indicated, the denatured samples were digested with endoglycosidase H or protein *N*-glycanase F (New England Biolabs) according to the manufacturer's instructions. All samples were then mixed with an equal volume of 2× Laemmli's buffer, denatured by heating (95 °C, 2 min), resolved by SDS-PAGE and transferred to PVDF membranes (Boname *et al.*, 2004). Membranes were blocked by pre-incubation in PBS/0.1 % Tween 20, 10 % non-fat milk, and then incubated with MHV-68-specific mAbs or biotinylated goat anti-GST pAb, followed by horseradish peroxidase (HRP)-conjugated rabbit anti-mouse IgG pAb (Dako Corporation) or HRP-conjugated streptavidin, and ECL substrate development (Amersham Biosciences).

### Immunofluorescence.

MHV-68-infected BHK-21 cells or transfected 293T cells were fixed in 4 % paraformaldehyde for 30 min, permeabilized with 0.1 % Tween 20 and then stained with MHV-68-specific mAbs (May *et al.*, 2005b). Fluorescent-tagged virions were visualized directly. For simultaneous staining of viral capsid and glycoprotein antigens, we used an IgG2a mAb against ORF65 (12B8) and an IgG1 mAb against gp150 (LSB11). Binding was then detected with Alexa 488-coupled IgG1-specific and Alexa 568-coupled IgG2a-specific pAb (Invitrogen). In some experiments, nuclei were counterstained with DAPI (4,6-diamidino-2-phenylindole). Fluorescence was visualized with a Leica confocal microscope.

### Flow cytometry.

Cells exposed to EGFP^+^ viruses were analysed directly for green-channel fluorescence. Transfected cells were trypsinized and stained with mAbs as described previously (Stevenson *et al.*, 2000). Detection was with fluorescein isothiocyanate-conjugated rabbit anti-mouse IgG pAb (DakoCytomation). Cells were analysed on a FACSort analyser by using CellQuest software (Becton Dickinson).

## RESULTS

### Identification of gB-specific neutralizing mAbs

**Fig. 1. f1:**
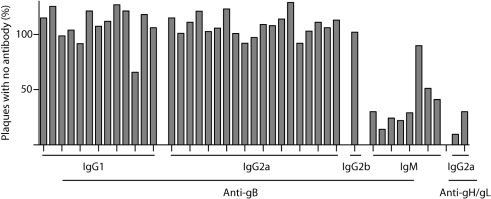
Identification of gB-specific neutralizing mAbs. MHV-68 (100 p.f.u.) was incubated with gB-specific mAb supernatants (2 ml) and then added to BHK-21 cell monolayers. Plaques were counted 4 days later and are expressed relative to the number of plaques with virus plus no antibody. The neutralizing gH/gL-specific IgG2a mAbs 7E5 and T2C12 were used as positive controls.

We previously derived seven MHV-68 gB-specific mAbs to identify gB as a virion component (Lopes *et al.*, 2004). These mAbs recognized virus-infected cells, but did not neutralize infectivity. In three subsequent fusions, we recovered another 75 gB-specific mAbs from MHV-68-infected mice. Again, none gave significant neutralization – 2 ml hybridoma supernatant reduced the infectivity of 100 p.f.u. MHV-68 by <50 % (data not shown). All gB-specific mAbs were selected for their capacity to recognize unfixed CHO cells expressing a GPI-linked form of the gB extracellular domain (Lopes *et al.*, 2004). These cells appear to reproduce every gB epitope that is available on MHV-68-infected BHK-21 cell surfaces (P. G. Stevenson, unpublished data). In a final fusion, seven of 41 gB-specific mAbs gave >50 % neutralization (Fig. 1).

The MHV-68-specific antibody response takes at least 3 months to reach a plateau level (Stevenson & Doherty, 1998), so all mAbs were derived from mice at least 3 months post-infection. Typically, 90–95 % were IgGs. However, all of the gB-specific neutralizing mAbs were IgMs (Fig. 1).

### Neutralizing mAbs target the gB N-terminal domain

**Fig. 2. f2:**
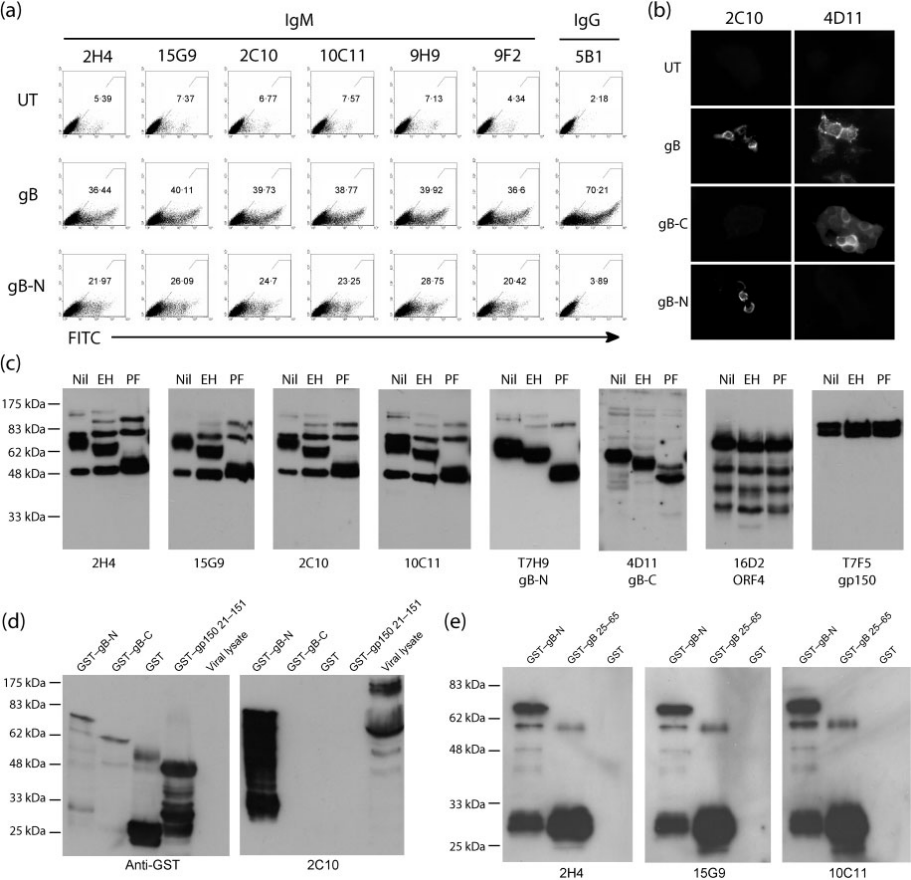
Recognition of N-terminal and C-terminal gB extracellular-domain fragments by virus-neutralizing IgMs. (a) 293T cells were left untransfected (UT) or transfected with a GPI-linked form of the gB extracellular domain (gB) or its N-terminal 423 aa (gB-N). Forty-eight hours later, all cells were stained with gB-specific mAbs as indicated. Each number is the percentage of cells in the positively staining region. 2H4, 15G9, 2C10, 10C11, 9H9 and 9F2 are all gB-specific neutralizing IgMs. 5B1 is a gB-specific non-neutralizing mAb that does not recognize gB-N. (b) 293T cells were transfected as in (a), but with an additional control of GPI-linked aa 423–698 of gB (gB-C), which was not detectable at the cell surface. After 48 h, the cells were fixed, permeabilized and stained with the gB-specific neutralizing mAb 2C10 or the gB-C-specific non-neutralizing mAb 4D11. The other neutralizing mAbs gave equivalent staining to 2C10. (c) MHV-68 virions were lysed, denatured and digested with no enzyme (Nil), endoglycosidase H (EH) or protein *N*-glycanase F (PF) to remove *N*-linked glycans. All samples were then resolved by SDS-PAGE and immunoblotted as indicated. T7H9 and 4D11 are specific for gB-N and gB-C, respectively. (d) gB fragments corresponding to its N-terminal and C-terminal extracellular-domain cleavage products were expressed as GST fusion proteins in *E. coli*. GST alone and GST fused to aa 21–151 of gp150 were used as specificity controls. Denatured proteins were immunoblotted for GST with a polyclonal serum or for gB with mAb 2C10. All of the gB-specific neutralizing IgMs gave the same results. (e) The N-terminal 40 aa of the mature gB extracellular domain were expressed fused to GST and immunoblotted with gB-specific IgM mAbs as in (d). Again, all of the gB-specific IgMs were indistinguishable.

The MHV-68 gB, like that of cytomegalovirus (Vey *et al.*, 1995), is cleaved by a furin-like enzyme as virions leave the cell (Lopes *et al.*, 2004). The predicted recognition site for furin in the MHV-68 gB is at aa 424–427. In order to identify which fragment of the gB extracellular domain contained its neutralization epitope(s), we tested the neutralizing IgMs for their recognition of each fragment expressed separately as a GPI-linked protein (Fig. 2a[Fig f2]). All of the neutralizing IgMs recognized the N-terminal gB fragment (aa 1–423) on transfected cell surfaces. None recognized the C-terminal part of the gB extracellular domain (aa 428–698), which did not reach the cell surface, but was detectable in the endoplasmic reticulum of transfected cells by the non-neutralizing IgG mAb 4D11 (Fig. 2b[Fig f2]). mAb 5B1 (Fig. 2a[Fig f2]) is equivalent in specificity to 4D11.

The neutralizing IgMs also immunoblotted gB in glycanase-treated virion lysates (Fig. 2c[Fig f2]). Their epitope(s) were therefore independent of gB folding, disulfide bonding or *N*-linked glycosylation. The minor 50 and 75 kDa bands detected by each IgM were probably non-specific, as they were detected by neither the gB-N-specific mAb T7H9 nor the gB-C-specific mAb 4D11. mAbs 16D2 (anti-ORF4) and T7F5 (anti-gp150) provided additional specificity controls. The IgMs also recognized N-terminal gB fused to GST and expressed in *E. coli* (Fig. 2d[Fig f2]). Their epitope(s) were therefore independent of *O*-linked glycosylation. The predicted size of the fusion protein was 74 kDa. The smaller products recognized on immunoblots were probably formed by degradation of the fusion protein from its C-terminal gB end rather than its N-terminal GST end, as they were recovered with glutathione-coated beads and the smallest product was still detected by an anti-GST pAb (Fig. 2d[Fig f2]). The molecular mass of GST is 26 kDa, so only a very small fragment of gB appeared to be required for recognition. This was confirmed by the recognition of a GST fusion protein containing only the N-terminal 40 aa of the mature gB (Fig. 2e[Fig f2]).

**Fig. 3. f3:**
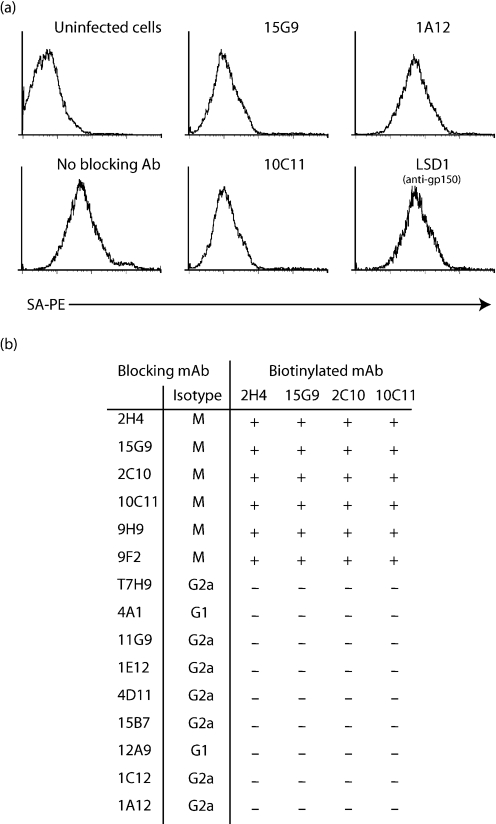
Mutual inhibition of binding between the gB-specific neutralizing IgM mAbs. MHV-68-infected BHK-21 cells (2 p.f.u. per cell, 18 h) were pre-incubated with gB-specific hybridoma supernatants, followed by a biotinylated gB-specific IgM as indicated. Binding of the biotinylated mAb to infected-cell surfaces was detected with fluorescent streptavidin and flow cytometry. An example assay is shown in (a), with mAb LSD1 as a non-gB-reactive isotype control, mAb 1A12 as a gB-reactive, non-neutralizing control and 15G9 as the biotinylated mAb. Summarized data for gB-specific mAbs are shown in (b). Blocking of biotinylated mAb staining is indicated by +; no effect on biotinylated mAb staining is shown by −.

The neutralizing IgMs also inhibited each other's binding to infected-cell surfaces (Fig. 3) and were indistinguishable by SDS-PAGE or immuno-electrophoresis (data not shown). We concluded that they were probably all derived from the same B-cell clone, presumably from just one mouse. The clone must have been large, as we were able to sample it multiple times from the pool of all gB-specific mAbs. However, our failure to identify an equivalent clone – or any other gB-specific neutralizing mAb – in four other fusions indicated that it was only rarely a major component of the response to MHV-68 infection.

### gB-directed neutralization operates at a post-binding entry step

**Fig. 4. f4:**
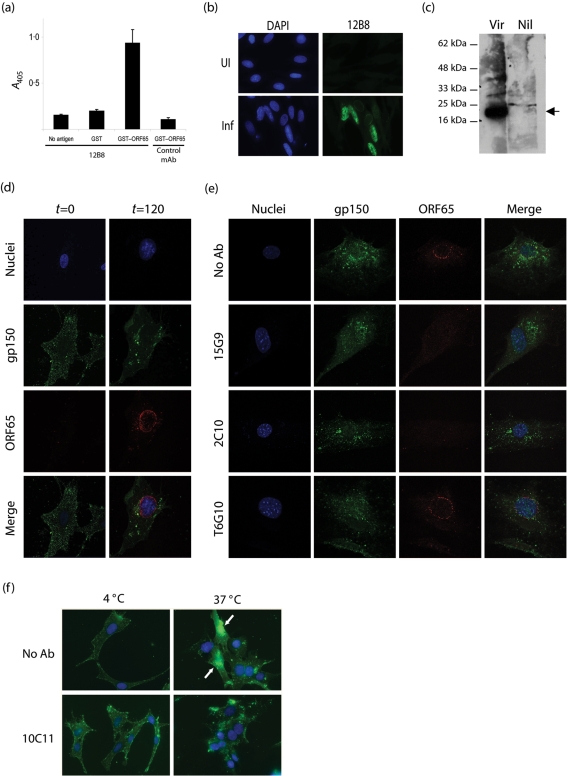
gB-directed neutralization blocks infection at a post-binding step. (a) ELISA plates were coated with no antigen, GST or GST fused to ORF65 and then incubated with mAb 12B8 or control mAb. Bound antibody was detected with alkaline phosphatase-coupled goat anti-mouse Ig and nitrophenylphosphate substrate. Mean±sd values are shown. (b) BHK-21 cells were infected (Inf) or not (UI) with MHV-68 (2 p.f.u. per cell, 18 h) and then fixed, permeabilized and stained with mAb 12B8. Nuclei were counterstained with DAPI. (c) BHK-21 cells were either left uninfected (Nil) or infected with MHV-68 (2 p.f.u. per cell, 18 h) (Vir). Cells were then lysed with 0.1 % SDS, sonicated and immunoblotted with mAb 12B8. (d) BHK-21 cells were exposed to MHV-68 for 2 h at 4 °C, then washed with PBS and either fixed (*t*=0) or incubated first for a further 2 h at 37 °C (*t*=120). All cells were stained for gp150 with mAb LSB11 and for ORF65 with mAb 12B8. Nuclei were counterstained with DAPI. (e) MHV-68 was pre-treated with mAbs as indicated, then added to cells (3 p.f.u. per cell, 37 °C). After 2 h, the cells were washed, fixed, permeabilized and stained as in (d). (f) MHV-68 was pre-treated or not with mAb 10C11 for 1 h, then incubated with cells for 4 h at either 4 or 37 °C before PBS washing. All cells were then fixed, permeabilized and stained for gp150 with mAb LSB11. Arrows indicate nuclear green fluorescence coming from viral EGFP expression in newly infected cells.

We used immunofluorescence and EGFP virus tagging to track MHV-68 entry into BHK-21 cells. We first derived a mAb specific for the MHV-68 ORF65 gene product – predicted to be a small capsid component (Fig. 4a–c[Fig f4]). mAbs from MHV-68-infected mice were screened by ELISA for specific recognition of ORF65 fused to GST (Fig. 4a[Fig f4]). The GST–ORF65-specific mAb 12B8 stained the nuclei of MHV-68-infected BHK-21 cells (Fig. 4b[Fig f4]) and immunoblotted an 18–22 kDa protein in virion lysates (Fig. 4c[Fig f4]) (the predicted molecular mass of the ORF65 gene product is 20 kDa).

The capsids of intact virions are surrounded by tegument and are therefore unlikely to be accessible to antibody. Accordingly, capsids were not seen immediately after adding virions to cells, but became visible when time was allowed for virion uncoating (Fig. 4d[Fig f4]). When virions were pre-treated with the gB-specific neutralizing mAbs 15G9 or 2C10, gp150 staining was not impaired (Fig. 4e[Fig f4]). Cell binding was therefore maintained. However, viral capsids failed to appear in significant numbers.

MHV-68 infects BHK-21 cells via endocytosis (Gill *et al.*, 2006). This is seen as a redistribution of incoming virion glycoproteins at 37 °C (Fig. 4d[Fig f4]). The redistribution still occurred when virions were pre-treated with a gB-specific neutralizing antibody (Fig. 4f[Fig f4]), implying that the virions had still been endocytosed. Neutralization was evident in the same experiment from a reduction in nuclear green fluorescence, i.e. a reduction in viral EGFP expression from the BAC cassette in newly infected cells. gB-directed neutralization therefore appeared to block a process linked closely to membrane fusion.

**Fig. 5. f5:**
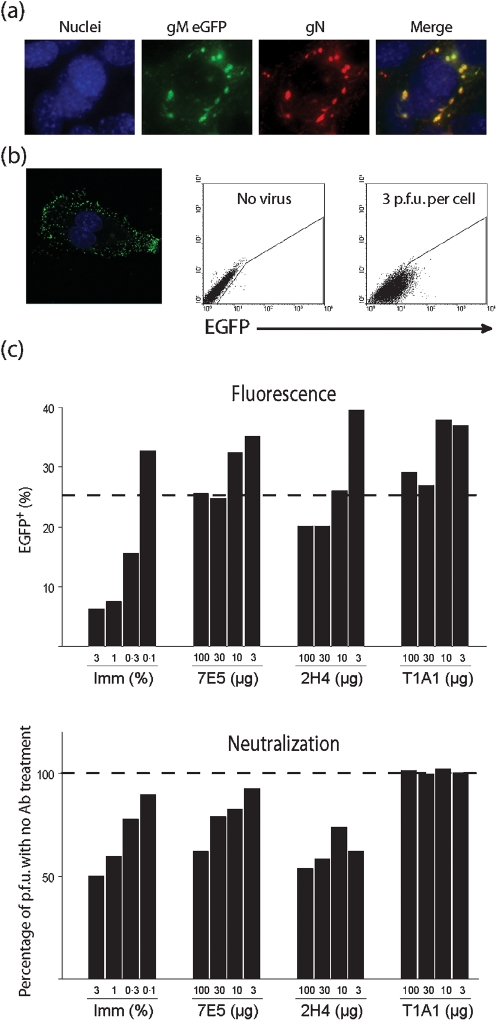
EGFP virus tagging reveals no obvious link between gB-directed neutralization and cell binding. (a) BHK-21 cells were infected (18 h, 2 p.f.u. per cell) with MHV-68 gM–EGFP (green) and then stained for gN with mAb 3F7. Nuclei were counterstained with DAPI. (b) BHK-21 cells were exposed to MHV-68 gM–EGFP (5 p.f.u. per cell, 1 h, 37 °C) and washed with PBS. EGFP fluorescence was visualized by confocal microscopy or flow cytometry. (c) MHV-68 gM–EGFP was mixed with MHV-68-immune mouse serum (Imm), a gH/gL-specific neutralizing mAb (7E5), a gB-specific neutralizing mAb (2H4) or a gp150-specific non-neutralizing mAb (T1A1). Each virus/antibody mixture was incubated with BHK-21 cells for parallel flow cytometry and plaque assay. The dashed lines indicate the limit of non-specific reductions in infectivity or EGFP transfer, as defined by the non-neutralizing mAb T1A1.

Flow cytometry using EGFP-tagged MHV-68 confirmed more quantitatively that gB-directed neutralization had little effect on cell binding (Fig. 5). For these experiments, we tagged the MHV-68 gM with C-terminal EGFP. The MHV-68 gN and gM form a disulfide-linked heterodimer (May *et al.*, 2005a). Thus, gM–EGFP fluorescence co-localized with gN-specific staining (Fig. 5a[Fig f5]). At early times after infection, incoming virions were visible as fluorescent dots on the plasma membrane that could also be detected by flow cytometry (Fig. 5b[Fig f5]). Virus neutralization by immune serum correlated with a considerable drop in cellular EGFP fluorescence (Fig. 5c[Fig f5]). Equivalent neutralization by anti-gH/gL or anti-gB mAbs gave little reduction in fluorescence. We consider a reduction to 25 % EGFP^+^ cells as non-specific, as this was seen with the non-neutralizing mAb T1A1. mAb 2H4 gave a marginally greater reduction. However, this has to be compared with a reduction to 5 % EGFP^+^ cells for equivalent neutralization by immune serum. It is possible – even likely – that the MHV-68 gB has a cellular ligand, but reduced cell binding did not appear to explain gB-directed neutralization.

### Comparison of gB-directed and gH/gL-directed neutralization

**Fig. 6. f6:**
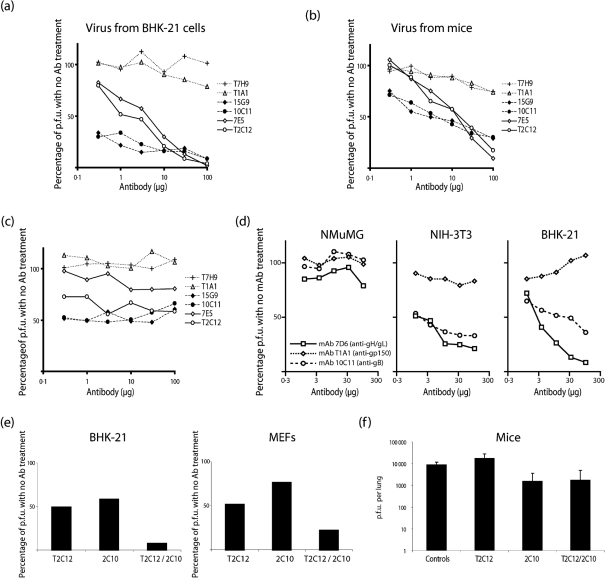
Neutralization of MHV-68 infectivity for different cell types with gB-specific and gH/gL-specific mAbs. (a) Wild-type MHV-68, grown in BHK-21 cells, was incubated with neutralizing mAbs against gB (15G9, 10C11) or gH/gL (7E5, T2C12), or non-neutralizing mAbs against gB (T7H9) or gp150 (T1A1). Each virus/antibody mixture was then titrated by plaque assay. (b) Wild-type MHV-68 was recovered from the lungs of infected mice and tested for neutralization with different mAbs as in (a). (c) The same virus/antibody mixtures as in (a) were titrated on MEFs rather than BHK-21 cells. (d) Virus was incubated with antibody (1 h, 37 °C) and then split for parallel titration on NMuMG epithelial cells, NIH-3T3 fibroblasts and BHK-21 fibroblasts. (e) Wild-type MHV-68 (200 p.f.u.) was incubated with 30 μg neutralizing mAbs against gH/gL (T2C12), gB (2C10) or both (T2C12/2C10). Each virus/antibody mixture was then titrated by plaque assay on BHK-21 cells or MEFs. (f) Wild-type MHV-68 (100 p.f.u.), grown in BHK-21 cells, was incubated with 100 μg neutralizing mAbs against gH/gL (T2C12), gB (2C10), both (T2C12/2C10) or no antibody (Controls). Each of five mice were infected intranasally with 1/100 of virus/antibody mixture (1 p.f.u.). Lungs were removed 6 days later and titrated for infectious virus by plaque assay.

gH/gL-directed MHV-68 neutralization notably requires relatively high levels of antibody (Gill *et al.*, 2006). By comparison, gB-directed neutralization gave a rapid reduction in infectivity as antibody levels increased, but less complete neutralization when antibody was saturating (Fig. 6a[Fig f6]) [note that Fig. 6(a) plots antibody concentration on a logarithmic scale]. Similar results were obtained with MHV-68 recovered directly from the lungs of infected mice (Fig. 6b[Fig f6]). Anti-gB or anti-gH/gL mAbs neutralized MHV-68 less well for MEF infection than for BHK-21 cell infection (Fig. 6c[Fig f6]) and failed to inhibit MHV-68 infection of NMuMG epithelial cells (Fig. 6d[Fig f6]). Virions coated completely with apparently neutralizing antibodies therefore still infected some cell types with a relatively high probability.

When anti-gB and anti-gH/gL mAbs were combined, the neutralization achieved was greater than with either alone (Fig. 6e[Fig f6]). We tested *in vivo* neutralization with single or combined neutralizing mAbs by adding saturating amounts of gB-specific and gH/gL-specific neutralizing mAbs to MHV-68 virions and then infecting mice intranasally with the equivalent of 1 p.f.u. each (Fig. 6f[Fig f6]). All of the mice became infected. Thus, at least some cells in the respiratory tract remained susceptible to infection by antibody-coated virions, i.e. NMuMG cells represented *in vivo* neutralization more faithfully than BHK-21 cells.

### High antibody avidity is crucial for gB-directed neutralization

**Fig. 7. f7:**
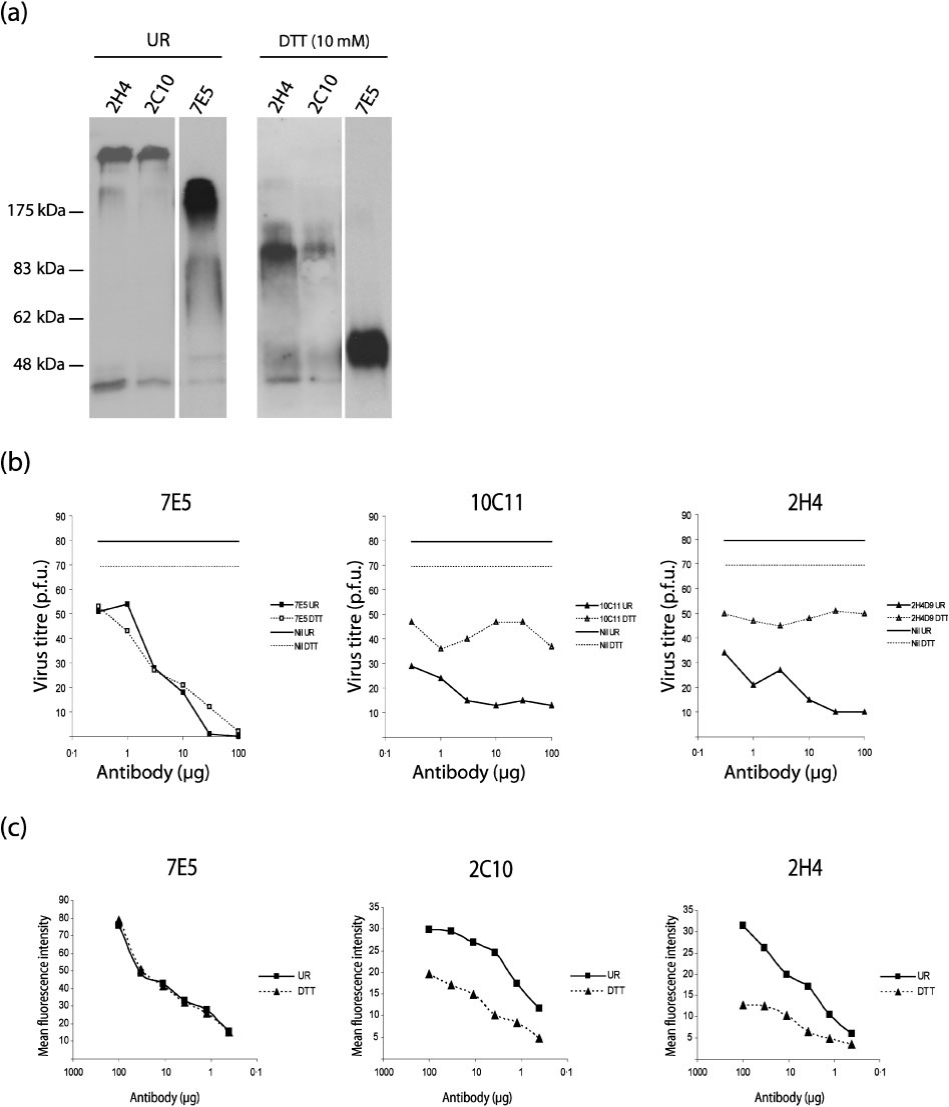
Disulfide bond reduction impairs gB-directed, IgM-mediated neutralization. (a) The gB-specific IgM mAbs 2H4 and 2C10 or the gH/gL-specific mAb 7E5 were reduced with 10 mM dithiothreitol (DTT) or not (UR), then denatured and immunoblotted for mouse Ig. (b) MHV-68 was incubated with equivalent reduced (DTT) or unreduced (UR) antibody preparations and then plaque-assayed on BHK-21 cells. Nil, No antibody. The data are representative of three experiments. mAbs 2C10, 10C11 and 2H4 all gave equivalent results. (c) Equivalent antibody preparations were used to stain by flow cytometry the surfaces of MHV-68-infected (2 p.f.u. per cell, 18 h) BHK-21 cells.

Secreted, pentameric IgM has a valency five times that of IgG. This high avidity helps to preserve suboptimal binding interactions in early antibody responses. However, MHV-68 does not induce sizeable long-term IgM responses – the virus is essentially a T cell-dependent antigen that elicits IgG (Stevenson & Doherty, 1999; Sangster *et al.*, 2000). The selection of a rare IgM mAb by the gB-directed neutralization screen therefore suggested that high avidity was functionally important. To test this, we reduced the gB-specific IgMs to monomers with 10 mM dithiothreitol (Fig. 7a[Fig f7]). This compromised neutralization severely (Fig. 7b[Fig f7]). By contrast, there was little effect on virus neutralization by the gH/gL-specific IgG2a mAb 7E5. The IgM monomers still bound to infected-cell surfaces (Fig. 7c[Fig f7]). They gave less fluorescence than the pentamers, but this presumably reflected that pentamers contain five times as many binding sites for secondary antibody detection. The binding of monomers and pentamers titrated similarly. Thus, although the pentameric structure of the IgMs was important for neutralization, it was not required for extracellular virion binding. It seemed more likely that high avidity helped to maintain virion binding after endocytosis.

## DISCUSSION

In order to establish the susceptibility of gammaherpesvirus virions to neutralization outside a purely *in vitro* context, we have begun to analyse the glycoprotein-specific antibody response of MHV-68-infected mice. The MHV-68 gB is an obvious candidate for neutralization: it is a highly conserved virion protein that is essential for infectivity and is equivalent to a reported neutralization target on other herpesviruses. However, although gB-specific mAbs could readily be derived, gB-specific neutralizing mAbs were rare. Even the most effective mAb reduced infectivity to a threshold value rather than to zero, was cell-type specific in its potency and was critically dependent on high avidity for even modest neutralization. Most tellingly, mAb combinations that appeared to neutralize quite well *in vitro* failed to prevent infection *in vivo*.

The requirements for complete herpesvirus neutralization *in vivo* may be distinct qualitatively as well as quantitatively from those for reducing infectivity *in vitro*. For example, blocking major receptor-binding interactions might make infection slower, but there is no particular reason to believe that time limits infection *in vivo*. Herpesviruses encode multiple cell-binding glycoproteins, and even heparan sulfate binding might be sufficient for endocytosis (Fuki *et al.*, 2000). Furthermore, the expression of immunoglobulin Fc receptors on mucosal surfaces *in vivo* (Spiekermann *et al.*, 2002) may allow virions incapable of cell binding still to infect via Fc receptor-mediated endocytosis. Direct *in vivo* neutralization tests therefore provide a crucial reality check for *in vitro* infectivity reductions.

As gB-directed neutralization acted close to the obligate infection step of viral membrane fusion, an alternative entry pathway seemed unlikely. The infectivity preserved with gB-directed neutralization probably reflected an appreciable chance of fusion occurring, even when every mAb binding site was occupied. One possibility is that not every gB molecule on virions was accessible. The MHV-68 gB N terminus – like that of several other gammaherpesviruses – is predicted to be heavily *O*-glycosylated, with nine potential glycan attachment sites in the first 30 aa alone. We have previously documented quite variable *O*-glycosylation of the MHV-68 ORF28 gene product (May *et al.*, 2005c). There may be similar heterogeneity in *O*-glycosylation near the gB N terminus, such that some gBs are bound poorly by antibody. Transformed cell lines cannot be considered representative of natural glycosylation, but MHV-68 propagated *in vivo* showed at least as much resistance to gB-directed neutralization as did that propagated in BHK-21 cells (Fig. 6b[Fig f6]). Such a limitation might also apply to the N-terminal RGD neutralization target on the KSHV gB (Akula *et al.*, 2002).

Alternatively, antibody-bound gBs may still retain an appreciable chance of participating in fusion, given the right cellular setting. MHV-68 infects both fibroblasts (Gill *et al.*, 2006) and epithelial cells (data not shown) via endocytosis, so any antibody blocking membrane fusion must remain attached in endosomes. Analogy with other viruses would suggest that MHV-68 membrane fusion results from energetically favourable conformational changes in viral glycoproteins at endosomal pH – essentially, viruses tap the cellular energy invested in endosomal acidification to drive membrane fusion. If the more mobile regions of viral fusion proteins are inaccessible, it may be difficult for antibodies to bind their targets strongly enough to block pH-driven conformation changes completely. The high avidity of IgM pentamers – which should be maintained in the oxidizing redox potential of endosomes (Austin *et al.*, 2005) – would allow them to compete more effectively, and this may explain why the best gB-specific neutralizing mAb was an IgM. Competition between antibody binding and glycoprotein conformation switching could also explain the differences in neutralization between cell types – NMuMG cells providing an environment more conducive to conformation changes than BHK-21 cells. Whatever the mechanistic explanation, the key practical point was that an NMuMG-like, susceptible target was available for incoming, antibody-bound virions to infect *in vivo*.

One way to overcome the difficulty of holding on to viral glycoproteins in endosomes would be to block receptor binding and thereby prevent endocytosis in the first place. This is probably the major mechanism by which mAbs neutralize small RNA viruses. It may also be the major mechanism by which immune sera neutralize herpesviruses (Fig. 5c[Fig f5]). However, as noted above, more robust herpesvirus neutralization is likely to be achieved by targeting viral membrane fusion in multiple ways. Encouragingly, combining gB-specific and gH/gL-specific mAbs was more effective than using either alone (Fig. 6). What must be appreciated is that herpesviruses have evolved over millions of years to protect themselves against neutralization, so overcoming this protection is unlikely to be easy.

## Figures and Tables

**Table 1. t1:** mAbs used in this study

**mAb**	**Target**	**Isotype**	**Neutralization**	**Reference**
15G9	gB*	IgM	Yes	This study
2C10	gB	IgM	Yes	This study
10C11	gB	IgM	Yes	This study
9H9	gB	IgM	Yes	This study
9F2	gB	IgM	Yes	This study
2H4	gB	IgM	Yes	This study
1C12	gB	IgG2a	No	This study
4D11	gB	IgG2a	No	This study
T7H9	gB	IgG2a	No	Lopes *et al*. (2004)
1A12	gB	IgG2a	No	This study
11G9	gB	IgG2a	No	This study
1E12	gB	IgG2a	No	This study
15B7	gB	IgG2a	No	This study
12A9	gB	IgG1	No	This study
4A1	gB	IgG1	No	This study
5B1	gB	IgG1	No	This study
7E5	gH/gL	IgG2a	Yes	Gill *et al*. (2006)
T2C12	gH/gL	IgG2a	Yes	Gill *et al*. (2006)
T6G10	ORF4†	IgM	No	This study
16D2	ORF4	IgG2a	No	Gill *et al*. (2006)
LSD1	gp150‡	IgM	No	This study
T1A1	gp150	IgG2a	No	de Lima *et al*. (2004)
LSB11	gp150	IgG1	No	This study
3F7	gN	IgG2a	No	May *et al*. (2005a)
12B8	ORF65§	IgG2a	No	This study

*mAbs recognized CHO-K1 cells expressing the extracellular domain of gB with a GPI anchor (Lopes *et al.*, 2004). †mAbs recognized CHO-K1 cells stably transfected with ORF4, and BHK-21 cells infected with wild-type, but not ORF4-deficient, MHV-68 (Adler *et al.*, 2000). ‡mAbs recognized L929 cells stably transfected with gp150, and BHK-21 cells infected with wild-type, but not gp150-deficient, MHV-68 (de Lima *et al.*, 2004). §See Fig. 4(a–c).
